# A Comparison of a Postal Survey and Mixed-Mode Survey Using a Questionnaire on Patients’ Experiences With Breast Care

**DOI:** 10.2196/jmir.1241

**Published:** 2011-09-27

**Authors:** Marloes Zuidgeest, Michelle Hendriks, Laura Koopman, Peter Spreeuwenberg, Jany Rademakers

**Affiliations:** ^1^Tranzo, Academic Research Centre for Health and Social CareTilburg UniversityTilburgNetherlands; ^2^Netherlands Institute for Health Services Research (NIVEL)UtrechtNetherlands

**Keywords:** Data collection, health care quality, consumer satisfaction, breast cancer, patient preferences, health care quality indicator

## Abstract

**Background:**

The Internet is increasingly considered to be an efficient medium for assessing the quality of health care seen from the patients’ perspective. Potential benefits of Internet surveys such as time efficiency, reduced effort, and lower costs should be balanced against potential weaknesses such as low response rates and accessibility for only a subset of potential participants. Combining an Internet questionnaire with a traditional paper follow-up questionnaire (mixed-mode survey) can possibly compensate for these weaknesses and provide an alternative to a postal survey.

**Objective:**

To examine whether there are differences between a mixed-mode survey and a postal survey in terms of respondent characteristics, response rate and time, quality of data, costs, and global ratings of health care or health care providers (general practitioner, hospital care in the diagnostic phase, surgeon, nurses, radiotherapy, chemotherapy, and hospital care in general).

**Methods:**

Differences between the two surveys were examined in a sample of breast care patients using the Consumer Quality Index Breast Care questionnaire. We selected 800 breast care patients from the reimbursement files of Dutch health insurance companies. We asked 400 patients to fill out the questionnaire online followed by a paper reminder (mixed-mode survey) and 400 patients, matched by age and gender, received the questionnaire by mail only (postal survey). Both groups received three reminders.

**Results:**

The respondents to the two surveys did not differ in age, gender, level of education, or self-reported physical and psychological health (all *Ps* > .05). In the postal survey, the questionnaires were returned 20 days earlier than in the mixed-mode survey (median 12 and 32 days, respectively; *P* < .001), whereas the response rate did not differ significantly (256/400, 64.0% versus 242/400, 60.5%, respectively; *P* = .30). The costs were lower for the mixed-mode survey (€2 per questionnaire). Moreover, there were fewer missing items (3.4% versus 4.4%, *P* = .002) and fewer invalid answers (3.2% versus 6.2%, *P* < .001) in the mixed-mode survey than in the postal survey. The answers of the two respondent groups on the global ratings did not differ. Within the mixed-mode survey, 52.9% (128/242) of the respondents filled out the questionnaire online. Respondents who filled out the questionnaire online were significantly younger (*P* < .001), were more often highly educated (*P* = .002), and reported better psychological health (*P* = .02) than respondents who filled out the paper questionnaire. Respondents to the paper questionnaire rated the nurses significantly more positively than respondents to the online questionnaire (score 9.2 versus 8.4, respectively; χ^2^_1_ = 5.6).

**Conclusions:**

Mixed-mode surveys are an alternative method to postal surveys that yield comparable response rates and groups of respondents, at lower costs. Moreover, quality of health care was not rated differently by respondents to the mixed-mode or postal survey. Researchers should consider using mixed-mode surveys instead of postal surveys, especially when investigating younger or more highly educated populations.

## Introduction

In the Netherlands, health care policy stresses regulated competition between health care providers [1]. Efforts are made to enhance the transparency of health care quality, to stimulate informed decision making among consumers, and to improve the performance of health care providers. Comparative information about the performance of health care providers is needed for consumers to make informed decisions. This comparative information can be gathered in different ways. One possibility is to ask a sample of patients about their actual experiences concerning quality of care provided by health care providers.

Measuring the quality of care from the patients’ perspective has been standardized in the Netherlands since 2006, using a new instrument called the Consumer Quality Index (CQ-index or CQI) [2]. CQI questionnaires are usually self-administered paper questionnaires (eg, CQI Rheumatoid Arthritis [3], CQI Breast Care [4]). Individual structured interviews are conducted in cases where a self-administered paper questionnaire is not feasible because of respondents’ visual, physical, or cognitive limitations (CQI Care for the Disabled [5], CQI Long-Term Care [6]). Postal surveys (with multiple reminders) and interviews are relatively expensive and time consuming. It would therefore be interesting to know whether other data collection methods can be applied in this field.

The Internet is increasingly considered to be an efficient medium for assessing quality of care from a patient’s perspective. In populations that already use the Internet, Internet surveys have been found to be a useful means of conducting research [7-9]. Efficiency gains are found in shorter response times and field costs reductions (50%–80%) [10-12]. In contrast to paper questionnaires, Internet questionnaires can contain various interactive features that allow complex skip patterns that are invisible to respondents, and the Internet allows validation of responses by using an instant feedback function while respondents are still online [12,13]. Consequently, the quality of data collected with an Internet survey is higher. Some Internet surveys have shown promising response rates (up to 94% in Web forums) [10,12,14]. The extreme response in Web forums can be explained by a probable selection bias in these studies. Those who participate in Web forums are most likely people who are familiar with the Internet and frequently use the Internet, leading to a higher response rate to Internet questionnaires. This high response rate has not been realized in other studies; response rates ranged from 17% to 70% [15]. In CQI research, the response rate to paper questionnaires varied from 20% to 79% with an average response rate of 55% [16]. One CQI study compared an Internet questionnaire with a paper questionnaire. The response rate to the Internet questionnaire (8%) was considerably lower than to the paper questionnaire (35%) [17,18]. To increase the response rate one can send a prenotification or reminders, give an incentive, or use short questionnaires. A salient subject of a questionnaire also increases the response rate [19].

The potential of Internet surveys should, however, be balanced against an equally large weakness. The Netherlands has the largest percentage of households with Internet access in the European Union, but there are still 1.2 million Dutch people (7.3% of the population) with no Internet access at home and 0.5 million Dutch people (3.1% of the population) who do not use the Internet [20]. People who use the Internet are more affluent, better educated, more often male, and younger than people who do not use Internet. Only a part of the population can thus be reached through the Internet [10,11,21]. To compensate for the selection of people in an Internet survey, a combination of data collection methods can be used such as combining an Internet questionnaire with a more traditional postal follow-up [19].

It is known that the way questionnaire are administered has an effect on answers of respondents (so-called mode effects). For example, telephone respondents were found to be more likely to rate health care positively and their own health status negatively than postal respondents [22,23]. This finding is similar to a study where telephone respondents provide more positive ratings than Web respondents [24]. Another example is that students who completed a Web-based questionnaire responded more favorably on different scales (such as college challenge and learning, education, and personal and social gains) than students who filled out a paper questionnaire [25]. It is suggested that computer anxiety affects participants’ responses. Moreover, biases could occur in the way people perceive and process questions presented on screen versus on paper. A study that tested the difference in test–retest reliability and internal consistency between Internet and paper versions of the SF-36, however, found little or no evidence for mode effects [26]. Knowing that these mode effects exist, it is important to investigate whether the answers of respondents in a postal and mixed-mode survey differ.

To examine whether a mixed-mode survey can be an alternative to postal survey, our research question is “What are the differences between a mixed-mode survey (Internet with paper follow-up) and a more traditional postal survey in terms of respondent characteristics, response rates and time, quality of data, costs, and mode effects?” The differences were examined within a sample of breast care patients who reported their experiences with health care using the CQI Breast Care questionnaire.

## Methods

### Sample

Data were collected within a larger study assessing the usability of CQI Breast Care [27]. For the mixed-mode survey, 200 patients with a benign abnormality and 200 patients with breast cancer were selected from the reimbursement files of seven Dutch health insurance companies. Inclusion criteria were (1) being older than 18 years and (2) having received breast care in the last 24 months. We used the same procedure to select 3955 patients who received the questionnaire by mail only as part of another study. Of these 3955 patients, we selected 400 patients (200 with breast cancer and 200 with benign abnormalities) for the comparison of the two surveys. These 400 patients were not randomly selected, but were matched by age and gender to the respondents in the mixed-mode survey.

### Data Collection

**Figure 1:**
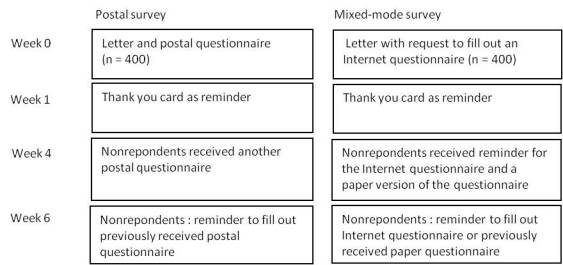
Mail-shots sent to the patients.

Patients received a letter from their health insurance company with the request to fill out a paper questionnaire (postal survey) or an Internet questionnaire with unique username and password (mixed-mode survey). A total of three reminders were sent and in both surveys nonrespondents received a paper version of the questionnaire in the third mail-shot. This data protocol was based on Dillman et al [28]. (See Figure 1 for detailed information on the mail-shots.) The data were collected in the Netherlands in the spring of 2008.

### Questionnaire

The CQI Breast Care contains items measuring the actual experiences of patients with breast examinations, surgery for breast cancer, other treatment, subsequent treatment, cooperation between health care providers, continuity of care, accessibility of care, and expertise of health care providers [4]. There are two versions of the CQ-index: one for patients with breast cancer (151 items) and one for patients with a benign abnormality (60 items). The questionnaire for patients with a benign abnormality is the same as the questionnaire for breast cancer, except that it does not contain questions about surgery and treatments. Both questionnaires have three scales in common, and the questionnaire for patients with breast cancer consists of 11 extra scales. Cronbach alpha for these scales varied between 0.74 and 0.93. Example items are presented in Table 1. The questionnaires additionally contain items on respondents characteristics (eg, age, education, ethnicity, and patient’s self-assessed physical and psychological health) and global ratings of health care providers general practitioner, hospital care in the diagnostic phase, surgeon, nurses, radiotherapy, chemotherapy, and hospital care in general). In the present study, we focused on the global ratings of the health care providers. These ratings ranged from 0 to 10, with a score of 0 indicating the worst possible health care or provider and a score of 10 indicating the best possible health care or provider. The respondents were asked to report their experiences in the last 24 months.

**Table 1:** Scales in the Consumer Quality Index Breast Care, their reliability (Cronbach alpha for internal consistency), and example items

Scale		Number of items	Alpha_1_^a^	Alpha_2_^b^	Total	Example of item
1	Conduct of professionals during breast examination	7	.90	.91	.90	How often did caregivers listen to you carefully?
2	Conduct of general practitioner	4	.91	.88	.90	How often did your general practitioner take you seriously?
3	Conduct of nurses	5	–	.87	.87	How often did nurses pay personal attention to you?
4	Conduct of surgeon	4	–	.89	.89	How often did the surgeon spend enough time with you?
5	Autonomy regarding treatment	4	–	.82	.82	How often did you get the chance to decide about your treatment?
6	Autonomy regarding follow-up treatment	2	–	.93	.93	How often were your specific wishes about follow-up treatment taken into account?
7	Conduct of professionals during radiotherapy	5	–	.88	.88	How often did you get the opportunity to ask questions about radiotherapy?
8	Information about radiotherapy	2	–	.78	.78	How often did you get enough information about radiotherapy?
9	Conduct of professionals during chemotherapy	4	–	.92	.92	How often did caregivers listen carefully to you?
10	Information about chemotherapy	4	–	.80	.80	How often did caregivers explain aspects of chemotherapy in a way that was easy to understand?
11	Cooperation	5	.91	.87	.89	How often did caregivers make good arrangements with each other?
12	Continuity of psychosocial care	3	–	.84	.84	Were you informed about the options for psychosocial care?
13	Continuity of physiotherapy	3	–	.74	.74	Were you assisted with a referral to physiotherapy?
14	Continuity of rehabilitation	3	–	.80	.80	Did you have as rapid access to a rehabilitation program as you wanted?

^a^ Questionnaire for patients with benign abnormality.

^b^ Questionnaire for patients with breast cancer.

### Statistical Analyses

#### Respondent Characteristics

To check whether our matching procedure was successful, we compared the selected patients within the two surveys on age and gender. Respondents were compared concerning age, level of education, self-reported physical and psychological health (Mann-Whitney test), and gender (χ^2^ test).

#### Response Rate and Time

Response rates were calculated as the number of valid received questionnaires divided by the number of patients in the starting sample. The response time was calculated as the number of days between the first letter (January 31, 2008) and the return date of the valid questionnaire. For the mixed-mode survey, the number of days between sending the paper questionnaire (February 28, 2008) and receiving the valid paper questionnaire was also calculated. The closing date of the data collection was April 1, 2008. A chi-square test was used to examine the differences in response rates between the two surveys because of the dichotomous variable (respondent/ nonrespondent). The differences in response time were determined using a Mann-Whitney test because the response time is a continuous variable.

#### Quality of Data

The percentage of items that were skipped while they needed to be answered (missing items) and the percentage of the items that were answered while they needed to be skipped (invalid answers) were calculated. The percentages were compared between the two surveys using a Mann-Whitney test because these percentages are continuous variables.

#### Total Costs

Expenses considered in cost calculations included setup costs (document layout, programming and testing of the questionnaire for each survey, and mailing supplies), field costs (postage, technical support, and project management staff), and scanning data costs (data entry of paper questionnaires). The costs per valid questionnaire received were calculated by dividing the total costs by the number of valid questionnaires received.

#### Mode Effects

We performed multilevel regression analyses to examine the mode effects. Multilevel regression analyses take into account the hierarchical structure of our data: individual patients (level 1) are nested within hospitals (level 2). The analyses were conducted using MLwiN version 2.02 software package (Centre for Multilevel Modelling, University of Bristol, Bristol, UK). Mode effects were examined by comparing the estimated mean scores on seven global ratings of general practitioner, hospital care in the diagnostic phase, surgeon, nurses, radiotherapy, chemotherapy, and hospital care in general using a chi-square test (*P* < .05 if χ^2^ > 3.8 and *P* < .001 if χ^2^ > 6.6). The mean scores were adjusted for the influence of age, education level, and self-reported health status of respondents.

 In addition, within the mixed-mode survey, we examined the differences in respondent characteristics, differences in response rates, and time and mode effects for respondents who filled out the Internet questionnaire and the paper questionnaire.

## Results

### Respondent Characteristics

Characteristics of the sample are presented in Table 2. Our matching procedure was successful since age and gender of the selected patients did not differ between the postal and mixed-mode survey. Patients with benign abnormalities were younger then patients with breast cancer (*P* < .001).

**Table 2:** Sample characteristics

		Postal survey	Mixed-mode survey	Mean difference	95% CI	*P* value
**Overall (n)**		400	400			
	Mean age (SD) years	55.5 (14.5)	55.5 (14.8)	–0.1	–2.1 to 1.9	.93
	Female	97.3% (389/400)	97.3% (389/400)	0.0	0.0	1.00
**Breast cancer (n)**		200	200			
	Mean age (SD) years	61.3 (12.7)	61.8 (12.9)	–0.4	–2.9 to 2.1	.77
	Female	99.0% (198/200)	99.0% (198/200)	0.0	0.0	1.00
**Benignabnormalities(n)**		200	200			
	Mean age (SD) years	49.5 (13.7)	49.3 (14.0)	0.2	–2.5 to 2.9	.89
	Female	95.5% (191/200)	95.5% (191/200)	0.0	0.0	1.00

Table 3 shows that also the characteristics of the respondents did not differ between the postal and mixed-mode survey.

**Table 3:** Respondents’ age, gender, level of education, and self-reported physical and psychological health

		Postal survey	Mixed-mode survey	Mean difference	95% CI	*P* value
**Overall (n)**		256	242			
	Mean age (SD), years	55.8 (13.5)	57.0 (13.6)	–1.2	–3.6 to 1.2	.32
	Female	97.7% (250/256)	97.5% (236/242)	0.1	–2.6 to 2.8	1.00
**Breast cancer (n)**		134	132			
	Mean age (SD), years	60.2 (12.4)	62.1 (12.4)	–1.9	–5.0 to 1.0	.26
	Female	98.5% (132/134)	99.2% (131/132)	0.7	–3.3 to 3.3	1.00
**Benignabnormalities(n)**		122	110			
	Mean age (SD), years	50.8 (13.1)	50.8 (12.4)	0.1	–3.2 to 3.4	.29
	Female	96.7% (118/122)	95.5% (105/110)	1.2	–3.7 to 6.2	.74
**Education level (n)**		251	232			
	Mean (SD)	4.4 (1.9)	4.6 (1.8)	–0.2	–0.6 to 0.1	.09
	Less than high school	41.1% (103/251)	31.0% (72/232)			
	High school graduated	20.3% (51/251)	25.0% (58/232)			
	Higher education	31.6% (79/251)	39.3% (91/232)			
	College degree	4.8% (12/251)	2.2% (5/232)			
	Other	2.4% (6/251)	2.6% (6/232)			
**Self-reported physical health (n)**		254	239			
	Mean (SD)	2.9 (0.8)	2.8 (0.9)	0.1	–0.04 to 0.3	.29
	Excellent	5.1% (13/254)	11.3% (27/239)			
	Very good	20.1% (51/254)	16.7% (40/239)			
	Good	55.5% (141/254)	55.6% (133/239)			
	Fair	17.3% (44/254)	13.4% (32/239)			
	Poor	2.0% (5/254)	2.9% (7/239)			
**Self-reported psychological health (n)**		255	239			
	Mean (SD)	2.6 (1.0)	2.6 (1.0)	0.1	–0.1 to 0.2	.40
	Excellent	16.9% (43/255)	18.4% (44/239)			
	Very good	18.4% (47/255)	23.0% (55/239)			
	Good	51.8% (129/255)	44.4% (106/239)			
	Fair	11.4% (32/255)	13.4% (32/239)			
	Poor	1.6% (4/255)	0.8% (7/239)			

Within the mixed-mode survey, differences were found between those who filled out the Internet questionnaire and those who filled out the paper questionnaire. Internet respondents were younger, were more often highly educated, and reported better psychological health compared with respondents who filled out the paper questionnaire (Table 4). Also, both paper and Internet respondents with benign abnormalities were younger than their counterparts with breast cancer (*Ps* < .001; not in table).

**Table 4:** Respondents’ characteristics within the mixed-mode survey: age, gender, level of education, and self-reported physical and psychological health

		Postal	Internet	Mean difference	95% CI	*P* value
**Overall (n)**		114	128			
	Mean age (SD), years	61.8 (14.0)	52.7 (11.6)	–9.1	–12.3 to –5.8	<.001
	Female	99.1% (113/114)	96.1% (123/128)	–3.0	–6.9 to 0.9	.22
**Breast cancer (n)**		68	64			
	Mean age (SD), years	67.9 (11.2)	56.0 (10.5)	–12.0	–15.7 to 8.2	1.00
	Female	98.5% (67/68)	100% (64/64)	1.5	–1.5 to 4.5	1.00
**Benignabnormalities(n)**		46	64			
	Mean age (SD), years	52.7 (12.9)	49.5 (11.9)	–3.2	–7.9 to 1.5	.18
	Female	100% (46/46)	92.2 % (59/64)	–7.8	–15.7 to 1.0	.07
**Education level (n)**		105	127			
	Mean (SD)	4.2 (1.8)	4.9 (1.8)	0.7	0.3 to 1.2	.002
	Less than high school	38.1% (40/105)	25.2% (32/127)			
	High school graduated	41.0% (43/105)	38.6% (49/127)			
	Higher education	18.1% (19/105)	29.9% (38/127)			
	University degree	0.0% (0/105)	3.9% (5/12)			
	Other	2.9% (1/105)	2.4% (3/127)			
**Self-reported physical health (n)**		112	127			
	Mean (SD)	2.9 (0.9)	2.7 (0.9)	–0.1	–0.4 to 0.1	.14
	Excellent	12.5% (14/112)	10.2% (13/127)			
	Very good	11.6% (13/112)	21.3% (27/127)			
	Good	55.4% (62/112)	55.9% (71/127)			
	Fair	17.9% (20/112)	9.4% (12/127)			
	Poor	2.7% (3/112)	3.1% (4/127)			
**Self-reported psychological health (n)**		112	127			
	Mean (SD)	2.7 (1.0)	2.4 (0.9)	–0.3	–0.5 to 0.03	.02
	Excellent	17.0% (19/112)	19.7% (25/127)			
	Very good	17.0% (19/112)	28.3% (36/127)			
	Good	46.4% (52/112)	42.5% (54/127)			
	Fair	18.8% (21/112)	8.7% (11/127)			
	Poor	0.9% (1/112)	0.8% (1/127)			

### Response Rates and Times

The response rate did not differ between the two surveys and was 64.0% (256/400 patients) for the postal survey and 60.5% (242/400 patients) for the mixed-mode survey (*P* = .31; Table 5). While the response rates of patients with breast cancer and of patients with benign abnormalities did not differ in the postal survey (134/200, 67.0% versus 122/200, 61.0%, respectively; *P* = .21), the response rate was significantly higher for patients with breast cancer than for patients with benign abnormalities in the mixed-mode survey (132/200, 66.0% versus 110/200, 55.0%, respectively; *P* = .02).

In the mixed-mode survey, 52.9% (128/242) of the respondents filled out the questionnaire online. The percentage of patients with benign abnormalities who filled out the questionnaire online was higher (64/110, 58%) than the percentage of patients with breast cancer (64/134, 49%). However, this difference was not significant (*P* *=* .13).

**Table 5:** Response rates for each survey and for patients with breast cancer or benign abnormalities

	Postal survey		Mixed-mode survey		Mean difference	95% CI	*P* value
Overall response	64.0%	256/400	60.5%	242/400	3.4%	–3.2% to 10.2%	.32
Breast cancer	67.0%	134/200	66.0%	132/200	1.0%	–8.3% to 10.3%	.83
Benign abnormality	61.0%	122/200	55.0%	110/200	6.0%	–3.7% to 15.7%	.23

**Figure 2:**
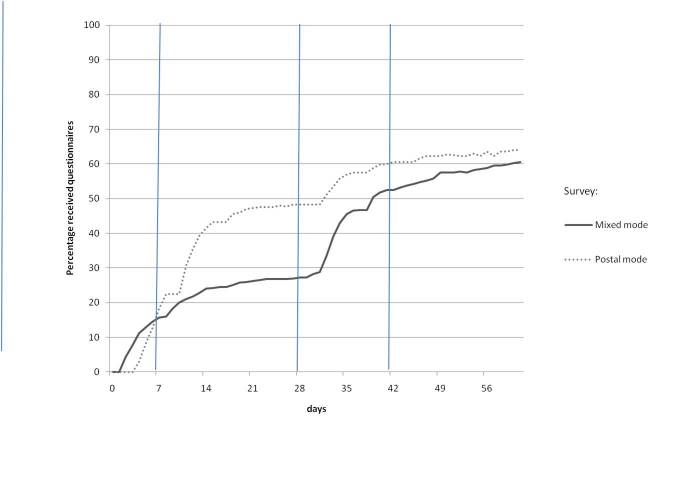
Percentage of received questionnaires by days after first mail-shot for the postal and mixed-mode surveys.

**Figure 3:**
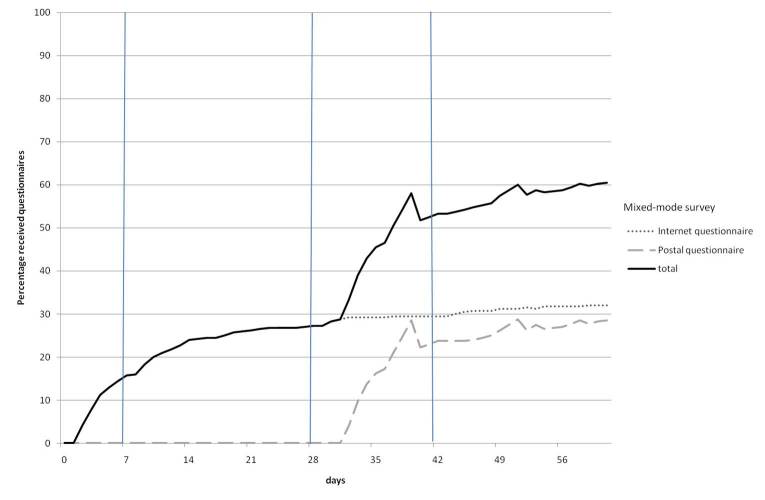
Percentage of received questionnaires by days after first mail-shot for the Internet and paper questionnaires within the mixed-mode survey.

Figure 2 and Figure 3 show the cumulative percentage of questionnaires received by days after the first mail-shot. The vertical lines in the graphs represent the reminders that were sent. In the postal survey, questionnaires were returned 20 days earlier than in the mixed-mode survey (*z* = –3.59, *P* < .001). The median number of days expired before the questionnaire was returned was 12 days (range 4–60 days) in the postal survey and 32 days (range 2–61 days) in the mixed-mode survey.

In the mixed-mode survey, the paper questionnaires were sent in week 4 (second reminder). The median number of days expired before these paper questionnaires were returned was 7 days (range 4–33 days). The median number of days expired before online questionnaires were filled out was 9 days (range 2–59 days). In other words, the longer response time in the mixed-mode survey was mainly caused by the group who did not respond using the Internet.

### Quality of Data

The mean percentage of missing items per question differed significantly between the two surveys (z = –3.08, *P* = .002): the mean percentage of missing items was lower in the mixed-mode survey than in the postal survey (5.04/150, 3.4% versus 6.60/150, 4.4%, respectively). In addition, the mean percentage of invalid answers was twice as high in the postal survey as in the mixed-mode survey (4.99/81, 6.2% versus 2.50/81, 3.2%, respectively; *z* = –3.68, *P* < .001).

### Costs

The costs per valid questionnaire returned were higher in the postal survey than in the mixed-mode survey (€25.8 versus €23.9 per valid questionnaire returned, respectively). Compared with the postal survey, the variable costs were reduced by 17% of the total costs in the mixed-mode survey, but the fixed costs were raised by 17% (Table 6).

**Table 6:** Fixed and variable costs per valid questionnaire returned

Costs		Postal survey		Mixed-mode survey	
	%		Cost (€)	%	Cost (€)
**Fixed costs**		41.5	10.7	58.4	14.0
	General	3.6	0.9	2.1	0.5
	Information technology (programming software, scanning, Internet questionnaire design)	35.0	9.0	53.8	12.9
	Processing results and making a data file	2.9	0.8	2.5	0.6
**Variable costs**		58.5	15.1	41.6	9.9
	Material (paper, envelopes)	1.4	0.4	0.9	0.2
	Printing (letters, survey, reminders)	26.3	6.8	21.6	5.2
	Preparing tasks (folding forms/questionnaires, thank you cards, filling envelopes)	4.2	1.1	4.6	1.1
	Response processing (opening envelope, checking, scanning data)	8.6	2.2	4.6	1.1
	Postal costs	17.9	4.6	9.9	2.4
**Total**		100.0	25.8	100.0	23.9

### Mode Effects

In Table 7, the mean scores on seven global ratings of different health care providers are presented. These mean scores have been corrected for hospital, age, level of education, and self-reported health status. The scores are relatively high, ranging from 8.3 to 9.0. Respondents in the postal survey gave the radiotherapist a score of 9.0 and the total care in the hospital a score of 8.3. The respondents in the mixed-mode survey rated the general practitioner and chemotherapy care the highest (score = 8.8) and gave care at the hospital in the diagnosis phase and hospital care a score of 8.4. We found no significant differences in global ratings between the two surveys.

**Table 7:** Mean scores on global ratings of different health care providers (corrected for hospital, age, education, and self-reported health status) for respondents to the postal survey and mixed-mode survey

		Postal survey			Mixed-mode survey			
Ratings of health care providers		n	Mean^a^	SE	N	Mean^a^	SE	χ^2^_1_
1	General practitioner	105	8.5	0.21	114	8.8	0.22	2.1
2	Hospital care in diagnostic phase	240	8.4	0.11	220	8.4	0.11	0.0
3	Surgeon^b^	85	8.9	0.19	102	8.5	0.20	2.6
4	Nurses^b^	75	8.7	0.19	77	8.7	0.20	0.1
5	Radiotherapy^b^	68	9.0	0.19	80	8.7	0.22	2.1
6	Chemotherapy^b^	41	8.9	0.24	50	8.8	0.23	0.2
7	Hospital care in general	239	8.3	0.11	222	8.4	0.14	0.5

^a^ Measured on an 11-point scale from 0 (worst possible) to 10 (best possible).

^b^ Only in breast cancer questionnaire.

Table 8 shows the differences in global ratings given by respondents to the paper and Internet questionnaires within the mixed-mode survey. The global rating of nurses differed significantly between these two groups: respondents filling out the paper questionnaire rated the nurses significantly more positively than respondents filling out the questionnaire online (score 9.2 versus 8.4, respectively; c^2^ > 3.8).

**Table 8:** Mean scores on global ratings of different health care providers (corrected for hospital, age, education, and self-reported health status) for respondents to the postal or Internet questionnaire within the mixed-mode survey

Ratings of health care providers		Paper questionnaire			Internet questionnaire			
		n	Mean^a^	SE	n	Mean^a^	SE	χ^2^_1_
1	General practitioner	48	8.7	0.25	66	8.7	0.23	0.0
2	Hospital care during diagnosis phase	96	8.4	0.16	124	8.3	0.16	0.3
3	Surgeon^b^	49	8.7	0.26	56	8.2	0.25	3.0
4	Nurses^b^	35	9.2	0.33	42	8.4	0.28	5.6
5	Radiotherapy^b^	38	8.7	0.28	43	8.5	0.26	0.3
6	Chemotherapy^b^	21	9.0	0.34	30	8.7	0.28	1.4
7	Care at hospital	98	8.4	0.16	124	8.3	0.16	0.3

^a^ Measured on an 11-point scale from 0 (worst possible) to 10 (best possible).

^b^ Only in breast cancer questionnaire.

## Discussion

This study examined whether a mixed-mode survey (Internet questionnaire with paper follow-up) is an alternative to the more traditional postal survey. The results showed that combining an Internet questionnaire with a paper follow-up improved the quality of data and was less expensive than a postal survey. However, the time before questionnaires were received was longer in the mixed-mode survey. No differences between the mixed-mode survey and postal survey were found concerning respondent characteristics, response rates, and global ratings of different health care providers.

 The findings showed that the characteristics of the respondents were the same for the two surveys. This means that mixed-mode surveys attract the same population as postal surveys. In total, 53% of respondents in the mixed-mode survey (128/242) filled out the questionnaire online. It appeared that in the mixed-mode survey Internet respondents were younger and more often highly educated and that they reported better psychological health than paper respondents. The younger people probably were more familiar with the Internet and were more likely to have access to the Internet than older people [11,21]. To overcome the problem of possible exclusion of the elderly and less highly educated, a mixed-mode survey should be chosen rather than an Internet survey [11,29].

The response rate was relatively high for both surveys (over 60%). In other CQI surveys, the response rates varied between 20% and 79% [16]. Perhaps the relatively high response rate is due the subject under study, namely abnormality of the breast. The response rate among women referred for mammography in another study was comparably high, both for the Internet (64%) and for the paper questionnaire (77%) [26]. Breast abnormality is a disease that has a huge impact on the emotional and physical quality of life of patients [30]. A review showed that saliency of the subject of questionnaires yields higher response rates [19]. Our results confirm the result of that review. In the mixed-mode survey, the response rate for patients with breast cancer was higher than the response rate for patients with benign abnormalities, even though the questionnaire for breast cancer was longer.

 The response time for the questionnaires to be returned was longer in the mixed-mode survey than in the postal survey. This effect was unexpected because using the Internet can reduce the time taken to return a questionnaire [10-12]. Both groups in the mixed-mode survey (paper and Internet respondents) responded relatively quickly (median number of days 7 and 9 days, respectively), but respondents with no access to or interest in the Internet questionnaire only responded after 4 weeks when the paper questionnaire was sent. The relatively quick response by postal respondents in the mixed-mode survey could be explained by the fact that respondents had already been informed about the study. Use of prenotification has been shown to shorten response times [19,31]. Another method to reduce the return time is sending the paper questionnaire out earlier.

Research has shown that an Internet survey results in more complete data compared with a postal survey [32]. This conclusion is confirmed in our study; the quality of data was higher in the mixed-mode survey than in the postal survey. One of the advantages of using the Internet for survey research is the technique of designing questionnaires so that complex skip patterns are invisible to respondents. As a consequence, the online questionnaire resulted in zero missing items and zero invalid answers (eg, answers to questions that had to be skipped). However, given the fact that some groups of people are underrepresented on the Internet (for instance, the elderly), conducting surveys through the Internet alone is not (yet) possible [11,21].

One of the key potential advantages of using the Internet over paper questionnaires is reducing costs. This study showed that the costs per returned questionnaire was €2 lower in the mixed-mode survey than in the postal survey. In the present study, the information technology costs were, however, relatively high for the mixed-mode survey. This was due to the need to program two applications, one for scanning the paper questionnaires and one for the Internet questionnaires. In the future, more costs can possibly be saved by using one and the same program for the different data collection methods within a mixed-mode survey. In addition, the variable costs per questionnaire were lower and the fixed costs per questionnaire were higher in the mixed-method survey than in the postal survey. Fixed costs per questionnaire can be reduced if a larger sample is taken, because the fixed activities are divided over the number of returned questionnaires. In other words, the larger the sample, the more money can be saved by using a mixed-mode survey.

Our study was the first to examine so-called mode effects between a mixed-method survey (Internet with paper follow-up) and postal survey. We found no differences between the two surveys concerning global ratings respondents gave to different health care providers. This is beneficial, because it implies that there is no bias in the scores that is a function of the manner of data collection. Other studies did find mode effects between the answers of telephone respondents and postal respondents [23], Internet respondents and telephone respondents [24], and Internet respondents and postal respondents [25,28,33]. One study investigated the differences between a postal and an Internet questionnaire, where a subset of the participants filled out also the alternative version (Internet and paper questionnaire, respectively). They found little or no evidence for a difference in test–retest reliability and internal consistency when they compared the Internet and paper versions of the questionnaire [26].

We did not ask why respondents in the mixed-mode survey did not fill out the questionnaire online. In one study among nonrespondents of an Internet questionnaire, the nonrespondents indicated that they did not have a computer or access to the Internet. Other reasons were having no experience with the Internet or not trusting the Internet [31]. This corresponds with findings by other researchers, who showed that factors influencing response times are privacy concerns and computer anxiety [19,28,33
